# Replication and Transmission of Influenza A Virus in Farmed Mink

**DOI:** 10.3390/v18010009

**Published:** 2025-12-20

**Authors:** Guojun Wang, Xiaoran Gao, Guoquan Zhang, Guohua Deng, Jianzhong Shi

**Affiliations:** 1 State Key Laboratory of Animal Disease Control and Prevention, Harbin Veterinary Research Institute, Chinese Academy of Agricultural Sciences, Harbin 150069, China; guojun.wang@imu.edu.cn (G.W.); zhguoquan@126.com (G.Z.); dengguohua01@caas.cn (G.D.); 2 The State Key Laboratory of Reproductive Regulation and Breeding of Grassland Livestock, College of Life Sciences, Inner Mongolia University, Hohhot 010070, China; 22108036@mail.imu.edu.cn; 3 Gansu Polytechnic College of Animal Husbandry & Engineering, Wuwei 733014, China

**Keywords:** mink, influenza virus, mixing vessel, transmission, seroconversion

## Abstract

Farmed mink are frequently exposed to circulating influenza A viruses (IAVs), as confirmed by viral isolation and serological evidence. Previous work reveals that naïve mink serve as susceptible hosts for both avian and human influenza strains, highlighting their potential role in influenza ecology. In this study, we investigated whether farmed mink naturally pre-exposed to H9 retain the capacity to serve as “mixing vessels” for reassorting human and avian IAVs. Our results demonstrate that they remain fully susceptible and permissive to infection by both avian H6N6 and human H1N1 influenza strains. Notably, efficient transmission of these viruses occurred among farmed mink, confirming their potential to sustain viral exchange. These findings indicate that farmed mink represent highly permissive hosts capable of facilitating reassortment between circulating human and avian IAVs. Given this risk, current mink farming practices may substantially increase the likelihood of a pandemic emergence. We therefore urge immediate revision, stringent enhancement, and rigorous enforcement of biosecurity protocols and active surveillance systems in fur farming operations.

## 1. Introduction

Influenza A viruses (IAVs) are segmented, single-stranded RNA viruses with a broad host range, including wild aquatic birds—their primary natural reservoir [1,2,3]. Spillover events into mammals (e.g., humans, bovines, swine, and mink) enable zoonotic transmission and viral diversification [4,5,6,7,8,9], historically leading to pandemics through reassortment (e.g., 1957 H2N2, 1968 H3N2, 2009 H1N1) [10,11,12,13]. Species such as swine, susceptible to avian and human IAVs, act as “mixing vessels,” generating novel strains with pandemic potential, as demonstrated by the 2009 H1N1 outbreak [12,13].

Mink (*Neovison vison*) are notably susceptible to diverse IAVs. Following the first documented outbreak of avian influenza A (H10N4) in Swedish mink farms (1984) [14,15,16,17], surveillance has identified frequent cross-species transmission events involving avian (H5N1, H5N6, H9N2) and mammalian-adapted (H1N1, H3N2) subtypes in global mink populations [1,18,19,20,21,22,23,24,25,26,27,28,29,30,31,32,33,34,35]. Their role as potential reservoirs is compounded by intensive global fur farming practices, which amplify zoonotic risks, as seen with SARS-CoV-2 transmission from mink to humans [36,37,38,39,40,41,42].

While previous challenge studies confirmed that naïve mink support productive infection and efficient transmission of diverse influenza A subtypes [4,43,44,45], sero-epidemiological evidence reveals extensive field exposure of farmed mink to both avian and human viruses [4,19,29,30,46,47,48,49]. To further evaluate the risks to human and animal health that farmed mink poses, we systematically assess whether farmed mink naturally pre-exposed to influenza retain their capacity as “mixing vessels” for human-avian reassortment. Through serological surveys and experimental assessments of the susceptibility and transmissibility of prevalent subtypes, we evaluated their potential to accelerate viral evolution and zoonotic spillover risk.

## 2. Materials and Methods

### 2.1. Ethical Approval and Biosafety

All procedures were approved by the Animal Ethics Committee of the Harbin Veterinary Research Institute (HVRI), Chinese Academy of Agricultural Sciences, and adhered to the Guide for the Care and Use of Laboratory Animals (Ministry of Science and Technology, China). Experiments involving live influenza viruses were conducted in an enhanced ABSL2+ facility at HVRI, certified by the Chinese Ministry of Agriculture and the China National Service for Conformity Assessment.

### 2.2. Influenza Antibody Detection by Hemagglutination Inhibition (HI) Assay

All mink were obtained from a commercial mink farm in Shangzhi City, Heilongjiang Province, China. Twelve-month-old female mink, weighing 1000–1200 g, were used in the study.

Blood was collected from the cranial vena cava of anesthetized mink (ketamine/xylazine: 20 and 1 mg/kg, respectively). Serum samples (n = 37) underwent receptor-destroying enzyme (Denka Seiken, Tokyo, Japan) treatment prior to HI antibody titration using 0.5% chicken erythrocytes. HI titers ≥ 20 were considered positive. Antigens included human pandemic A/Sichuan/1/2009 [SC/09 (H1N1)] and avian viruses (A/duck/Chongqing/S4101/2010 [DK/10 (H3N2)], A/duck/Jiangxi/S21055/2012 [DK/12 (H4N2)], H5N1 influenza vaccine Re-6 bearing the HA and NA genes from A/duck/Guangdong/S1322/2010 [DK/10 (H5N1)], A/chicken/Guangdong/1311/2010 [CK/10 (H6N6)], A/duck/Zhejiang/C2046/2012 [DK/12 (H9N2)], and A/duck/Hunan/S4013/2011 [DK/11 (H11N9)]).

### 2.3. Sialic Acid Receptor Detection in Mink Tissues

Respiratory tissues from three euthanized mink (ketamine/xylazine: 100 and 5 mg/kg, respectively) were paraffin-embedded, sectioned (5 μm), and mounted on APS-coated slides (Matsunami Glass, Osaka, Japan). Sections were deparaffinized (xylene), rehydrated (ethanol), and probed with FITC-labeled *Sambucus nigra* (SNA) lectin (250 μL; Vector Laboratories, Newark, CA, USA) for α2,6 glycans, or Biotinylated *Maackia amurensis* (MAA) lectin (250 μL; Vector Laboratories) for α2,3 glycans. After overnight incubation (4 °C) and TBS washes, sections were incubated with Alexa Fluor 594-conjugated streptavidin (2 h, RT; Molecular Probes, Eugene, OR, USA), counterstained with DAPI (Dojindo, Kumamoto, Japan), and visualized using a Nikon ECLIPSE TE300 fluorescence microscope (Nikon, Tokyo, Japan). Images were captured with an Olympus DP70 camera (Olympus Corporation, Tokyo, Japan).

### 2.4. Viral Infection and Transmission Studies

For viral infection, three minks were inoculated intranasally (i.n.) with 10^6^ EID_50_ of each tested virus (1 mL total; 500 μL/nostril) under anesthesia (ketamine/xylazine: 20 and 1 mg/kg). Tissues (nasal turbinate, accessory, tonsil, trachea, lung, liver, kidney, spleen, apophysis mamillaris, intestine, brain) were harvested on day 4 post inoculation (p.i.). Tissues were homogenized, centrifuged (3000× *g*, 10 min), and supernatants titrated in 10-day-old embryonated eggs (n = 3 per dilution) using allantoic inoculation. HA titers were determined with 0.5% chicken erythrocytes, as per [5]. Briefly, the EID_50_ was determined by inoculating 10-day-old embryonated eggs with 10-fold serial dilutions of virus in PBS via the allantoic route (100 µL/egg). Eggs were incubated at 37 °C for 48 h, with infection endpoints defined by HA assay. EID_50_/mL was calculated using the Reed and Muench method.

For the direct transmission studies, three farmed minks were inoculated i.n. with 10^6^ EID_50_ of CK/10 (H6N6) and housed in separated cages placed inside an isolator. Three H6-naïve animals were introduced into a different cage with an infected animal 24 h later. For the studies on respiratory droplet transmission, groups of three mink were inoculated i.n. with 10^6^ EID_50_ of SC/09 (H1N1) and housed in specially designed cages inside an isolator, as described previously [5,13,50]. Twenty-four hours later, three H1-naïve animals were placed in an adjacent cage (4 cm away), separated by a double-layered net divider. The ambient conditions for these studies were set at 20–22 °C and a relative humidity of 30–40%. The airflow in the isolator was horizontal with a speed of 0.1 m/s. For the respiratory droplet transmission studies, the airflow direction was from the inoculated animals to the exposed animals. Nasal washes were collected at 2-day intervals, beginning on day 2 p.i. (day 1 post exposure) and titrated in eggs. Sera were collected on day 21 p.i. and tested for the presence of HI antibody. To prevent inadvertent physical transmission of virus by the investigators, the contact minks were always handled first, and gloves, implements, and napkins on the work surface were changed between animals. Clinical signs, temperature, and body weight were recorded daily for all animals.

### 2.5. Statistical Analysis

The relative receptor distributions (quantification of fluorescence intensity) of the organs were analyzed via one-way ANOVA (GraphPad Prism 9.5). Receptor distribution was quantified in Fiji software (Fiji Is ImageJ, v2.17.0) by masking organ regions of interest. After background subtraction, one-way ANOVA (α = 0.05, two-tailed) with Dunnett’s post hoc test compared all groups to the nasal tip.

## 3. Results

### 3.1. Seroprevalence of Antibodies Against Influenza Viruses in Farmed Mink

Mink have previously been known to be susceptible to human and avian influenza A virus infection [1,22,23,51]. Here, we conducted a serological survey to assess the prevalence of influenza virus exposure to farmed minks. The overall seroprevalence of influenza viruses in the collected mink samples was 89.2% (33/37 [positive for H9N2 HI]), and negative for the rest of the subtypes, including human H1N1 and avian H3N2, H4N2, H5N1, H6N6, and H11N9 (Table 1).

### 3.2. Analysis of the Receptor Distribution in the Respiratory System of Mink

**Figure 1:**
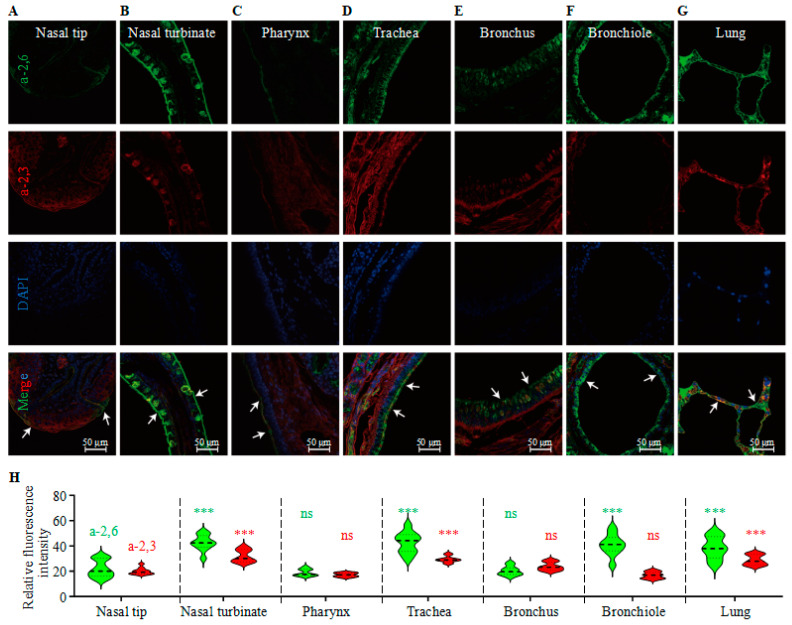
Distribution of influenza virus glycan receptors in the mink respiratory tract. (**A**–**G**) Immunofluorescence staining of α2,3- and α2,6-linked SA receptors. Tissues were stained with *Maackia amurensis* lectin II (MAA-II, red; α2,3 glycans) and *Sambucus nigra* lectin (SNA, green; α2,6 glycans) and counterstained with DAPI (blue). The arrows highlight receptor localization in the (**A**) nasal tip, (**B**) nasal turbinate, (**C**) pharynx, (**D**) trachea, (**E**) bronchus, (**F**) bronchiole, and (**G**) lung. (**H**) Quantitative analysis of receptor density. Fluorescence intensity of α2,3 and α2,6 glycans in 10 non-overlapping ROIs (per tissue) was measured using Fiji software. Data are presented as mean ± SEM; statistical significance was determined by one-way ANOVA (***, *p* < 0.001 vs. nasal tip; ns, not significant).

Influenza virus infects cells by first binding to sialic acid (SA) receptors on the cell surface; there are mainly two types of SA receptors: α2,6-linked SAs (also known as the human-type receptor) and α2,3-linked SAs (also known as the avian-type receptor) [5,13,50,52]. We therefore investigated the types of sialic acid receptors in different tissues of mink, and we found α2,3 and α2,6 glycans in mink samples from the nasal tip, nasal turbinate, pharynx, trachea, bronchus, bronchiole, and lung. The α2,6 glycans (green channel) was highly expressed in the nasal turbinate, trachea, bronchus, bronchiole, and lung, whereas the α2,3 glycans (red channel) showed significant expression in the nasal turbinate, trachea, bronchus, and lung. Specifically, the bronchiole predominantly expressed α2,6 glycans; the nasal turbinate, trachea, bronchus, and lung contained both α2,3 and α2,6 glycans; and the pharynx and nasal tip exhibited relatively low levels of both glycans (Figure 1). This receptor distribution allows both human and avian viruses to bind to the respiratory tissues of mink.

### 3.3. Replication and Transmission of Circulating Avian Influenza Virus in Farmed Mink

**Figure 2:**
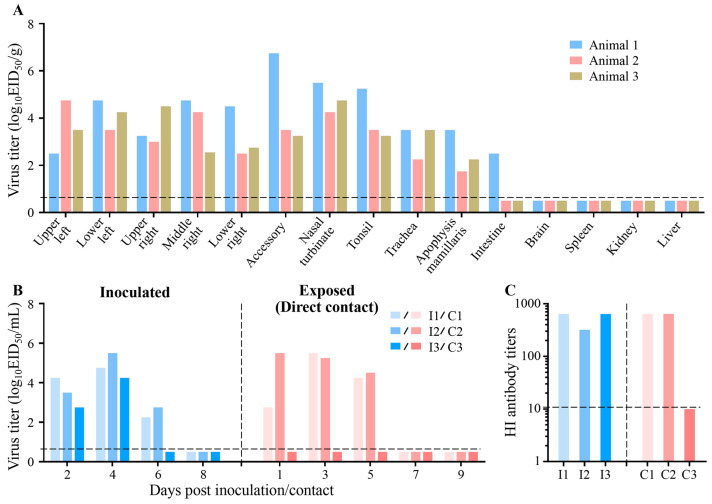
Replication and transmission of avian IAV in farmed mink. (**A**) Viral replication of CK/10 (H6N6) in farmed mink. Three minks were inoculated i.n. with 10^6^ EID_50_ of CK/10 (H6N6). On day 4 p.i., the nasal turbinate, accessory, tonsil, trachea, lung, liver, kidney, spleen, apophysis mamillaris, intestine, and brain were harvested for virus titration using EID_50_ assays in embryonated eggs. Each colored bar represents log_10_ virus titers (EID_50_/g) in an individual animal. (**B**) Direct contact transmission of CK/10 (H6N6) in farmed mink. Three H9-seropositive minks (donors) were inoculated with 10^6^ EID_50_ of CK/10 (H6N6). On day 1 p.i., one H9-pre-exposed mink (contact, “C”) was co-housed with each donor (“I”). Nasal washes were collected every 48 h and titrated in eggs. (**C**) Seroconversion in farmed mink following avian influenza virus infection or exposure. Serum was tested for H6-specific antibodies HI assay on day 21 p.i. Horizontal dashed lines indicate the lower limits of detection.

H6 AIV is widely prevalent in wild and domestic aquatic and terrestrial avian species throughout the world and is likely to play an important role in the ecology of influenza viruses. Previous studies have demonstrated that naïve mink are susceptible to circulating avian and human influenza viruses [4,43,44,45]. To assess the susceptibility of farmed mink to heterosubtypic influenza viruses, H9-seropositive minks were inoculated i.n. with an avian-origin H6N6 IAV. Infected minks exhibited transient clinical signs, including pyrexia (peaking at day 2 p.i.; Figure S1A), sneezing, and nasal discharge, alongside a maximum body weight loss of 6.4% (Figure S1B). The H6N6 virus replicated efficiently throughout the respiratory tract and intestinal tissues, as quantified by EID_50_ (Figure 2A). High titers of infectious virus (peak: 10^5.5^ EID_50_/mL) were shed in nasal washes for up to day 6 p.i., confirming robust upper respiratory tract replication (Figure 2B).

Our previous study showed that H6 viruses isolated from live poultry markets in southern China are able to transmit efficiently to direct contact animals, but fail to transmit via aerosol droplets [50]. To assess the transmissibility of avian influenza A viruses among farmed mink, we inoculated i.n. three H9-seropositive minks with 10^6^ EID_50_ of test virus and housed them individually in cages. Twenty-four hours later, another three H9-seropositive minks were placed in each cage. Evidence of transmission was based on the detection of virus in the nasal wash and on seroconversion at the end of the 3-week observation period. As shown in Figure 2B, in the CK/10 (H6N6)-inoculated groups, virus was detected in the nasal washes of all three inoculated minks as well as of two of the three contact animals. Seroconversion occurred in all inoculated animals and in the contact animals that were virus-positive, but did not change the HI titer of the previously existing H9 antibody (Figure 2C). These results indicate that other subtypes of avian influenza virus are still able to transmit efficiently among the pre-exposed contact mink.

### 3.4. Replication and Transmission of Circulating Human Influenza Virus in Farmed Mink

**Figure 3:**
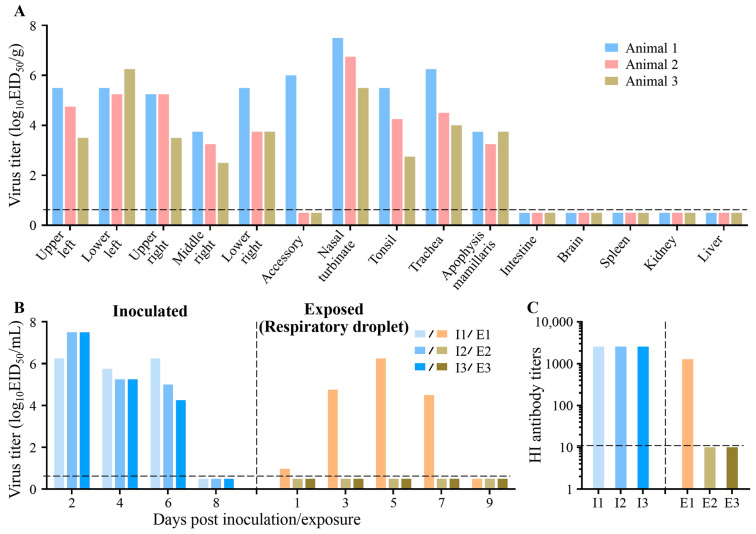
Replication and transmission of human IAV in farmed mink. (**A**) Viral replication of SC/09 (H1N1) in farmed mink. Three minks were inoculated i.n. with 10^6^ EID_50_ of SC/09 (H1N1). On day 4 p.i., the nasal turbinate, accessory, tonsil, trachea, lung, liver, kidney, spleen, apophysis mamillaris, intestine, and brain were harvested for virus titration using EID_50_ assays in embryonated eggs. Each colored bar represents log_10_ virus titers (EID_50_/g) in an individual animal. (**B**) Respiratory droplet transmission of SC/09 (H1N1) in farmed mink. Three H9-seropositive minks (donors) were inoculated with 10^6^ EID_50_ of SC/09 (H1N1). On day 1 p.i., one H9-pre-exposed mink (respiratory droplet, “E”) was housed in a wire frame cage 4 cm from each donor (“I”). Nasal washes were collected every 48 h and titrated in eggs. (**C**) Seroconversion in farmed mink following human influenza virus infection or exposure. Serum was tested for H1-specific antibodies HI assay on day 21 p.i. Horizontal dashed lines indicate the lower limits of detection.

IAVs currently circulating in humans include H3N2 and the pandemic H1N1 strain (responsible for the 2009 global outbreak). The spillover of human IAVs into other animals raises biosecurity and infection prevention and control issues. Previous research has demonstrated that naïve farmed mink are highly susceptible to and efficiently transmit circulating pandemic H1N1 influenza viruses [4]. To assess the susceptibility of pre-exposed mink to heterosubtypic influenza viruses, H9-seropositive minks were inoculated i.n. with pandemic H1N1 IAV (SC/09 (H1N1)). Clinically, the mink infected with pandemic H1N1 virus exhibited transient signs of pyrexia (Figure S1C), sneezing, and nasal discharge. The SC/09 (H1N1) virus caused the mink to experience maximum body weight loss of 12.54% (Figure S1D). The virus was exclusively detected throughout the respiratory tract (Figure 3A). Virus shedding up to day 6 p.i. was evident from infected mink for H1N1 virus, with a peak virus titer of 10^6.25^ EID50/mL based on nasal washes (Figure 3B).

Owing to their high transmissibility, the pandemic H1N1/2009 viruses rapidly disseminated worldwide and soon became established as a zoonotic–anthroponotic pathogen. To assess the transmissibility of human influenza A viruses (SC/09(H1N1)) among farmed mink, H9-seropositive minks were placed 4 cm away, in groups of three in separate wire cages, from infected mink 24 h post infection. By day 4 p.i., aerosol transmission was observed in one of three recipient mink, as confirmed by viral detection and subsequent seroconversion (Figure 3B,C). These results indicate that human influenza virus is still able to transmit among the pre-exposed mink.

## 4. Discussion

Our study demonstrates that influenza-pre-exposed mink retain their capacity to serve as “mixing vessels” for avian and human influenza A viruses (IAVs), reinforcing the zoonotic risks associated with intensive mink farming. Despite serological evidence of frequent H9N2 exposure to farmed mink [1,19], these animals remained fully susceptible to heterosubtypic strains, including avian H6N6 and human-origin H1N1 viruses. Both strains replicated efficiently in the respiratory tract and exhibited sustained transmission among pre-exposed mink, confirming their potential to facilitate reassortment between avian and human IAVs. These findings parallel observations in swine, a well-documented mixing vessel, but highlight a critical gap in biosecurity oversight for mink farming, underscoring their role as underappreciated reservoirs for viral evolution.

Host species susceptible to both human and animal influenza viruses may act as “mixing vessels” for viral reassortment, a phenomenon well-documented in pigs [49,53]. Within the *Mustelidae* family, ferrets are widely recognized as an optimal animal model for influenza research due to their close physiological resemblance to humans, including lung anatomy and SA receptor distribution (α2,6 glycans and α2,3 glycans) [54,55]. Our findings, supported by previous research [19], confirm that mink similarly express both α2,3 and α2,6 glycans in their respiratory tracts. The dual receptor tropism observed in mink underscores their potential as intermediate hosts for IAVs, heightening concerns about their role in zoonotic transmission and viral evolution. Notably, the anatomical distribution of IAVs’ receptors aligns with their documented susceptibility to both avian and human viral strains [1,56]. This receptor promiscuity mirrors findings in humans, where α2,6 glycans dominates the upper respiratory tract, while α2,3 glycans persists in lower regions, suggesting a shared mechanism for cross-species viral adaptation. Notably, the co-localization of both receptors in the nasal turbinate, trachea, and lung may facilitate viral reassortment or host-switching events, as seen in other mammalian models.

In influenza virus transmission research, ferrets and guinea pigs are the most frequently used animal models, though each has distinct advantages and limitations. While guinea pigs are highly susceptible to infection and efficient transmitters, their key drawback is their minimal clinical symptoms, even when infected with highly pathogenic avian influenza (HPAI) strains lethal to mice and ferrets [57,58]. By contrast, ferrets are considered the gold standard for studying human airborne transmissibility due to their close resemblance to humans in clinical presentation, pathogenesis, and immune response, as well as their natural susceptibility to influenza A and B viruses, enabling controlled studies on transmission dynamics and illness progression [13]. However, their high cost and limited commercial availability pose practical challenges [59]. As an alternative, mink—which express human-like influenza virus receptors—are increasingly recognized as a viable model due to their susceptibility to infection, widespread farming (making them cost-effective and readily available), and potential for generating statistically robust data at lower expense. These findings suggest that mink could serve as a valuable mammalian model for assessing the public health risks posed by human and avian influenza viruses.

The high seroprevalence of H9N2 antibodies (89.2%) in farmed mink confirms frequent exposure to avian influenza, consistent with previous reports of mink susceptibility and the wide spread of H9N2 IAVs in avian populations [5,58,60]. While no other tested subtypes (human H1N1, avian H3N2, H4N2, H5N1, H6N6, and H11N9) were detected in this cohort, earlier studies in China documented variable seropositivity rates (e.g., 20–47.5% for H9N2; 6.7% for H5N1) [1]. Mammalian influenza infections typically arise from direct contact with contaminated materials (e.g., raw infected meats, wild-bird droppings) [1,36]. The dominance of H9N2 in our study suggests regional viral circulation, though the absence of other subtypes may reflect sampling limitations or temporal fluctuations in exposure.

Experimental studies using influenza-naïve mink have evaluated their susceptibility to diverse influenza A viruses (IAVs) [4,43,44,45]. Notably, a recent study demonstrated that circulating human IAVs (H3N2 and H1N1/pdm09) efficiently transmit to 100% of co-housed mink via respiratory contact [4]. Similarly, avian-origin H3N2 and H7N2 viruses have shown transmission competence in mink under direct contact conditions [43,44,45]. However, sero-epidemiological data indicate widespread IAV exposure in farmed mink populations [4,19,29,30,46,47,48,49], raising a critical question: does the persistent viral circulation sustain an ongoing risk for zoonotic spillover or reassortment events? Although our findings confirm direct contact transmission of avian influenza viruses (H6N6: 2/3) and limited airborne transmission of human-origin pdmH1N1 (1/3) in pre-infected mink, the observed transmission rates were lower than those reported in previous studies [13,50]. This discrepancy may stem from partial cross-protection conferred by pre-existing immunity, mediated by broadly reactive antibodies and T-cell responses [34,35,36]. Nevertheless, these results underscore the capacity of mink to support avian and human influenza virus transmission, reinforcing their role as potential ‘mixing vessels’ for novel viral strain generation. Continuous risk assessment and enhanced mink surveillance are therefore essential to monitor viral evolution and spillover threats.

## Figures and Tables

**Table 1:** Hemagglutination inhibition (HI) antibody titers against influenza A viruses in farmed mink serum.

Serum No.	SC/09 (H1N1)	DK/10 (H3N2)	DK/12 (H4N2)	DK/10 (H5N1)	CK/10 (H6N6)	DK/12 (H9N2)	DK/11 (H11N9)
1	<10	<10	<10	<10	<10	640	<10
2	<10	<10	<10	<10	<10	640	<10
3	<10	<10	<10	<10	<10	640	<10
4	<10	<10	<10	<10	<10	1280	<10
5	<10	<10	<10	<10	<10	640	<10
6	<10	<10	<10	<10	<10	640	<10
7	<10	<10	<10	<10	<10	640	<10
8	<10	<10	<10	<10	<10	640	<10
9	<10	<10	<10	<10	<10	80	<10
10	<10	<10	<10	<10	<10	640	<10
11	<10	<10	<10	<10	<10	<10	<10
12	<10	<10	<10	<10	<10	1280	<10
13	<10	<10	<10	<10	<10	<10	<10
14	<10	<10	<10	<10	<10	320	<10
15	<10	<10	<10	<10	<10	1280	<10
16	<10	<10	<10	<10	<10	20	<10
17	<10	<10	<10	<10	<10	1280	<10
18	<10	<10	<10	<10	<10	1280	<10
19	<10	<10	<10	<10	<10	640	<10
20	<10	<10	<10	<10	<10	640	<10
21	<10	<10	<10	<10	<10	640	<10
22	<10	<10	<10	<10	<10	80	<10
23	<10	<10	<10	<10	<10	640	<10
24	<10	<10	<10	<10	<10	640	<10
25	<10	<10	<10	<10	<10	640	<10
26	<10	<10	<10	<10	<10	640	<10
27	<10	<10	<10	<10	<10	1280	<10
28	<10	<10	<10	<10	<10	640	<10
29	<10	<10	<10	<10	<10	80	<10
30	<10	<10	<10	<10	<10	320	<10
31	<10	<10	<10	<10	<10	<10	<10
32	<10	<10	<10	<10	<10	1280	<10
33	<10	<10	<10	<10	<10	40	<10
34	<10	<10	<10	<10	<10	1280	<10
35	<10	<10	<10	<10	<10	160	<10
36	<10	<10	<10	<10	<10	<10	<10
37	<10	<10	<10	<10	<10	40	<10

Notes: HI tests were performed with 0.5% chicken red blood cells; serum was pre-treated with receptor-destroying enzyme (RDE) to remove nonspecific inhibitors; HI titer ≥ 20 (positive), <10 (negative; below detection limit).

## Data Availability

The original contributions presented in the study are included in the article, and further inquiries can be directed towards the corresponding author/s.

## Supplementary Materials

The following supporting information can be downloaded at https://www.mdpi.com/article/10.3390/v18010009/s1. Figure S1: Body temperature and weight changes in farmed mink.
