# Supplementary material for: Replication and Transmission of Influenza A Virus in Farmed Mink
Source: Viruses. 2025 Dec 20;18(1):9. doi: 10.3390/v18010009 (PMC12846398; doi:10.3390/v18010009)
Supplement: Supplementary file 1 [file viruses-18-00009-s001.zip › viruses-4053170-supplementary.pdf]

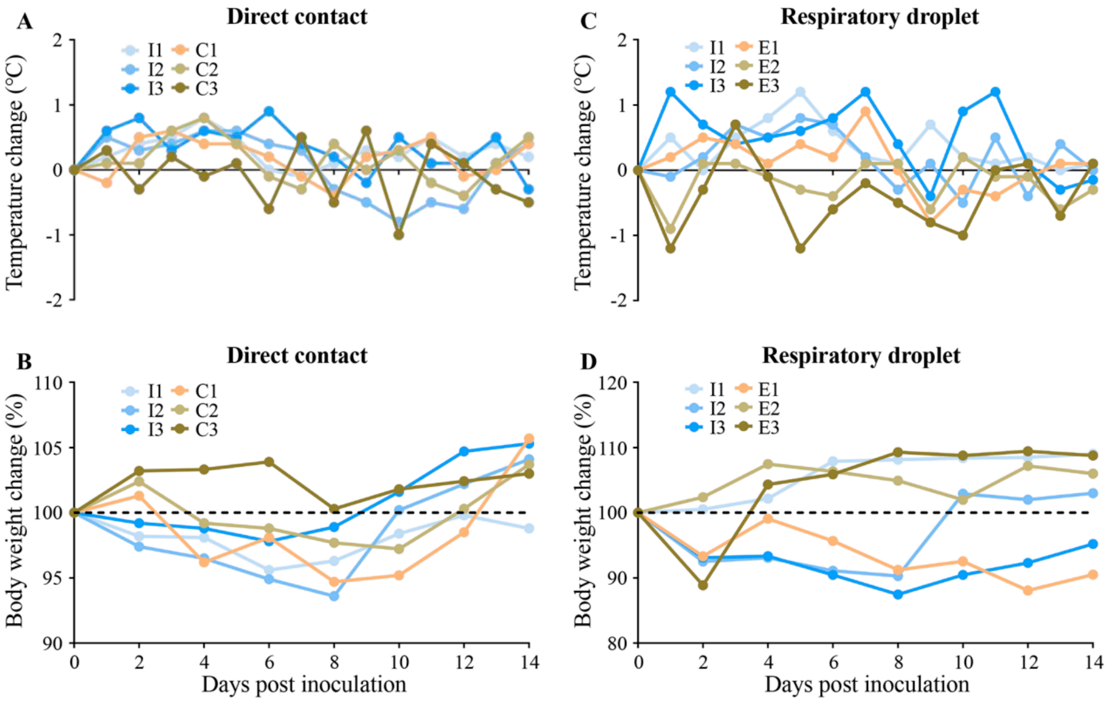

Figure S1. Body temperature and weight changes in farmed mink. (**A** and **B**) Direct contact transmission of CK/10 (H6N6) in farmed mink. (**C** and **D**) Respiratory droplet transmission of SC/09 (H1N1) in farmed mink. (**A**) Body temperature changes. (**B**) Body weight changes. (**C**) Body temperature changes. (**D**) Body weight changes.
